# Knowledge mapping and current trends of immunotherapy for prostate cancer: A bibliometric study

**DOI:** 10.3389/fimmu.2022.1014981

**Published:** 2022-10-27

**Authors:** Weibo Zhong, Zefeng Shen, Yongxin Wu, Xiangming Mao, Jianqiu Kong, Weixia Wu

**Affiliations:** ^1^ Department of Urology, Zhujiang Hospital, Southern Medical University, Guangzhou, Guangdong, China; ^2^ Department of Urology, Sun Yat-sen Memorial Hospital, Sun Yat-sen University, Guangzhou, Guangdong, China; ^3^ Guangdong Provincial Key Laboratory of Malignant Tumor Epigenetics and Gene Regulation, Guangzhou, China

**Keywords:** prostate cancer, immunotherapy, bibliometric analysis, sipuleucel-T, immune check inhibitor

## Abstract

**Background:**

Prostate cancer (PCa) is the second most common malignancy in men worldwide. Growing evidence substantiates the important role of immunotherapy in human tumors. Given that immunotherapy is often unsatisfactory on PCa, many studies have been conducted on PCa immunotherapy to improve treatment efficacy. However, no relevant bibliometric study of PCa immunotherapy has hitherto been reported. A bibliometric analysis was performed to evaluate the global scientific production of PCa immunotherapy research and characterize the development trends for future studies in this article.

**Methods:**

The publications related to PCa immunotherapy were extracted from the Web of Science Core Collection. The contribution and co-occurrence relationships of countries/regions, institutions, journals, references, authors, and keywords were assessed and visualized by VOSviewer and CiteSpace to identify research hotspots and potential future trends.

**Results:**

A total of 3,583 publications related to PCa immunotherapy from 1999 to 2021 were collected. The results of annual publications and citations exhibited a steady increase over the past 22 years. The National Cancer Institute in the USA published far more papers during the study than any institute. Accordingly, the USA had the most publications (*n* = 1,954, 54.54%). Gulley, James L. had the most number of published papers, and Small, Eric J. was the most co-cited authors in this field. *Cancer Immunology Immunotherapy* was the most productive journal, with 145 publications on PCa immunotherapy. Keyword cluster and keyword burst analyses showed that research in PCa immunotherapy shifted from “t cell infiltration” and “sipuleucel t” to “immune checkpoint inhibitor”, “CTLA-4”, and “PD-L1 expression”.

**Conclusion:**

PCa immunotherapy has attracted much attention, reflected by the increasing number of annual publications and citations. Much emphasis has been placed on exploring the complex immunogenicity and tumor microenvironment for PCa and identifying the patient population who can benefit from immunotherapy. Combining immune checkpoint inhibitors with other therapeutic options and cancer vaccines represents the future development trends in PCa immunotherapy.

## Introduction

Prostate cancer (PCa) is the most common malignant tumor of the male genitourinary system and ranks second in new cases and fifth in mortality among male malignant tumors worldwide (1). An increasing body of evidence suggests that localized PCa is curable by radical prostatectomy or radiotherapy, but the prognosis of advanced or metastatic PCa is poor (2, 3). Unlike other types of malignancies, androgen is an important risk factor for promoting the progression of PCa. Androgen deprivation therapy (ADT) has been developed accordingly, including castration and androgen receptor-targeted therapy. Chemotherapy remains the first-line treatment option for advanced PCa (including taxane, mitoxantrone, and platinum) (4). Both ADT and chemotherapy inevitably lead to drug resistance (5, 6). Once patients with PCa progress to the metastatic castration-resistant PCa (mCRPC) stage, the overall survival (OS) is significantly reduced (7), emphasizing the need to identify new treatments to effectively control the disease progression and prolong the OS of this patient population.

Immunotherapy is a novel treatment reported to be efficient in various human tumors (8–10). PCa is considered an immunologically “cold” tumor, characterized by low mutation load, with multiple immune escape mechanisms, complex immunogenicity, and tumor microenvironment (TME) without active response for immunotherapy (11, 12). Although many immunotherapeutic methods have been applied to PCa, including cancer vaccine, immune checkpoint inhibitor (ICI), and chimeric antigen receptor (CAR) T cells, the autologous cellular vaccine sipuleucel-T remains the only approach approved by the U.S. Food and Drug Administration (FDA) for mCRPC in 2010 (13). ICI for immunotherapy is mainly based on cytotoxic T lymphocyte antigen 4 (CTLA-4) (ipilimumab) or programmed cell death protein 1 (PD-1)/programmed cell death ligand 1 (PD-L1) (pembrolizumab) inhibition and yields excellent performance against various malignancies, such as melanoma, lung cancer, and urothelial carcinoma (8–10). However, the results of these drugs used alone or in combination are unsatisfactory for PCa (14–16). In recent years, attempts have been made to identify PCa patients that may derive benefit from ICI and the optimal disease stage for immunotherapy. CAR T cells have shown promising efficacy in hematologic malignancies but failed in solid tumors with immunosuppressive TME, dominated by high levels of multiple immune inhibitory factors, including transforming growth factor (TGF)-β. A first-in-human phase I trial showed that it was feasible and generally safe for castration-resistant, PCa-directed CAR T cells armored with a dominant-negative TGF-β receptor (17). To sum up, although the past two decades have witnessed significant progress in PCa immunotherapy, the role of immunotherapy in PCa warrants further exploration.

To our knowledge, no study has systematically evaluated PCa immunotherapy through bibliometric analysis. The bibliometric analysis takes the document system and document metrological characteristics as the research object, and uses quantitative research methods to analyze the document distribution, relationship, change, and progress in a certain field (18–20), performed by visual tools, such as VOSviewer (21, 22) and CiteSpace (23). In this study, we applied bibliometric analysis to analyze the countries, institutions, journals, authors, references, and keywords of PCa immunotherapy from 1999 to 2021. This study sought to reveal the current situation and research trends for PCa immunotherapy.

## Methods

### Data retrieval strategy

**Figure 1:**
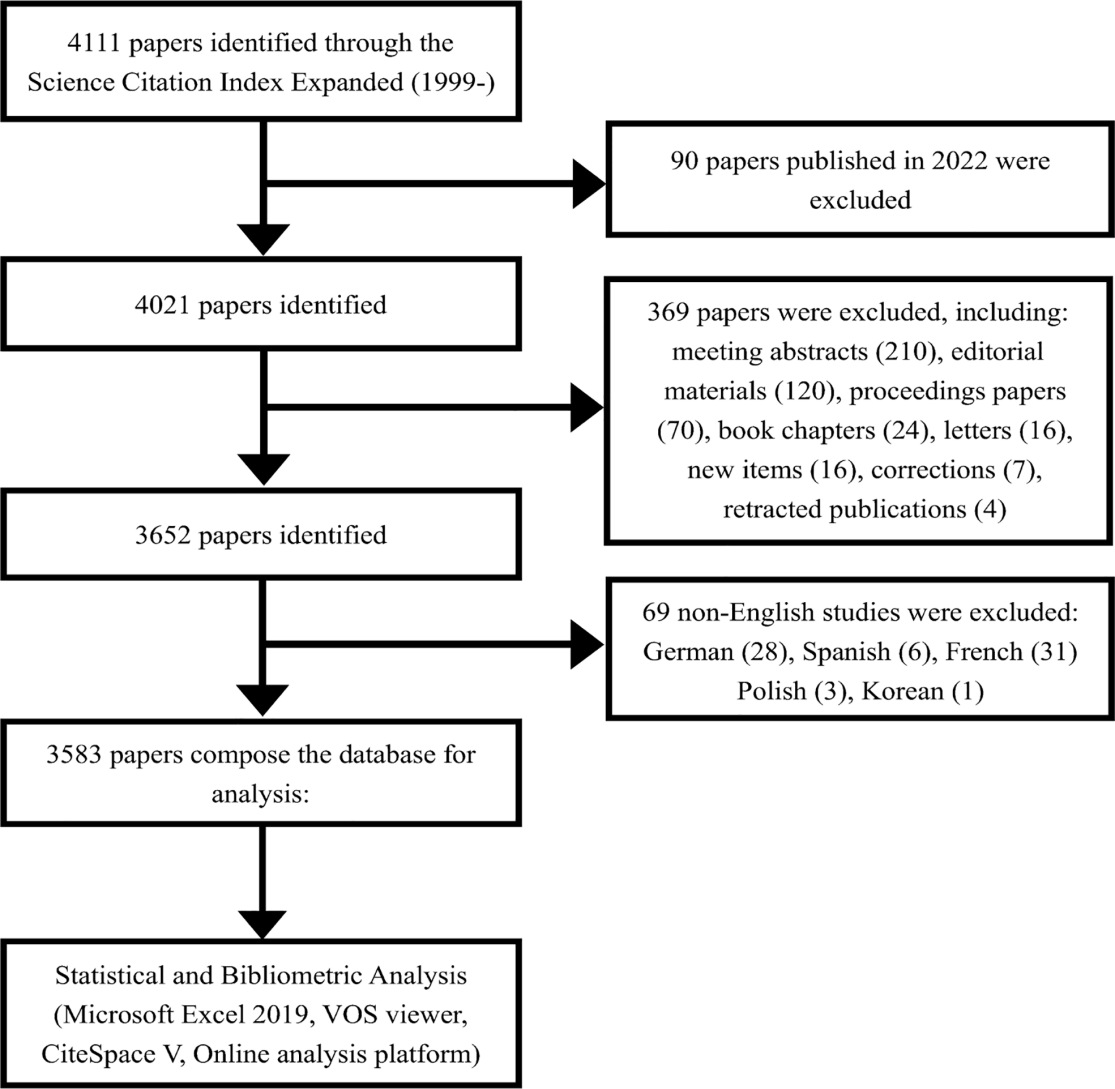
Flowchart of the literature screening process.

Literature on PCa and immunotherapy from 1999 to 2021 was searched using the Science Citation Index-Expanded (SCIE) of the Web of Science Core Collection (WoSCC). WoSCC is one of the most professional and authoritative citation-based databases with powerful indexing functions, which not only contains the basic information including authors, institutions, countries/regions, funding agencies, and author keywords but also includes the reference information (24–26). The search was conducted until 24 April 2022. The language was limited to English, and only original articles and reviews were collected. The following keywords were entered for the database retrieval using Boolean search operators: TS = (immunotherapy OR immunotherapies OR immunotherapeutic) AND TS = (prostate OR prostatic) NEAR/1 (cancer* OR tumor* OR tumour* OR oncology OR neoplasm* OR carcinoma*). The detailed screening process is shown in **Figure 1**.

### Statistical and bibliometric analysis

Microsoft Office Excel 2019 (Microsoft, Redmond, WA, USA) was used for the descriptive statistical analysis and for generating graphs. Meanwhile, a polynomial regression model was used to analyze the trends of annual citations and publications through Microsoft Office Excel 2019.

Bibliometric visualization was performed by VOSviewer and CiteSpace V. VOSviewer (Version 1.6.16) is a widely used software in bibliometrics developed by van Eck and Waltman (27). In this study, VOSviewer was used to perform the co-citation analysis of references/journals, co-occurrence analysis of author keywords, and co-authorship analysis of countries/institutions/authors. CiteSpace V (Version 5.8.R3) is also a popular visual tool (28–30) for co-authorship analysis of institutions, citation burst analysis of keywords, and timeline view analysis of co-cited references in this study. The parameter settings for CiteSpace were as follows: time span = 1999–2021, slice length = 1, selection criteria = top 50 per slice, node types = (reference, institution, keyword), pruning = (minimum spanning tree, pruning sliced networks), and visualization = cluster view-static.

## Results

### Analysis of publications and citations showed an overall increase in PCa immunotherapy research

**Figure 2:**
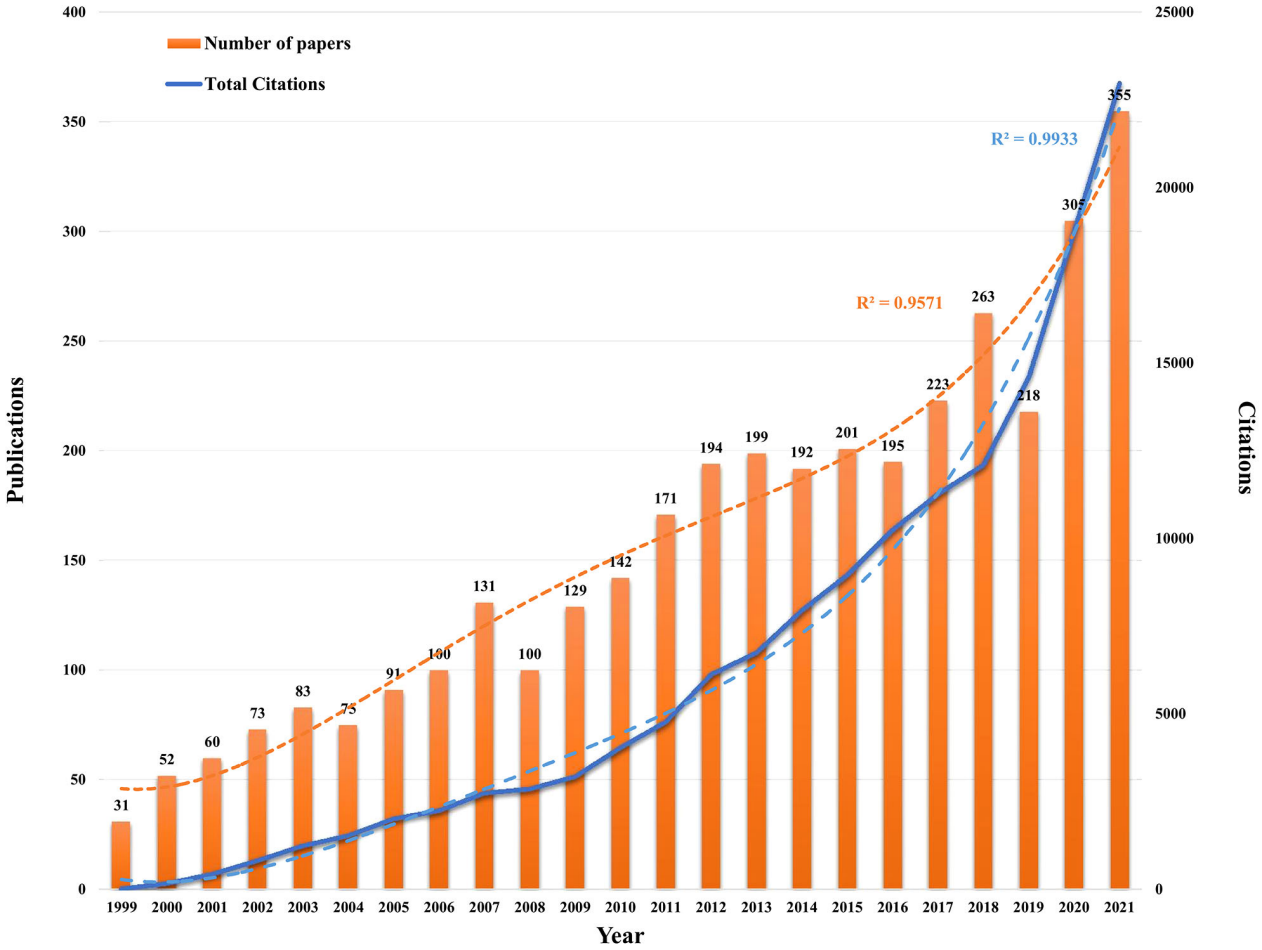
Annual trend and polynomial fitting curve of published articles and citations in the field of PCa immunotherapy in the database of WoSCC from 1999 to 2021.

According to our screening process, 3,583 publications related to PCa immunotherapy were included in this study by retrieval from the WoSCC database from 1999 to 2021. Overall, the annual number of publications and citations on PCa immunotherapy exhibited an upward trend (**Figure 2**). The model fitting curve of publications and citations suggested a significant increasing trend (*R* ^2^ = 0.9571 and 0.9933). The number of papers rapidly increased from 2017, with over 200 publications per year. Although there was a decrease in publication in 2019, the annual number of publications was more than 300 in 2020 and 2021. Significant increases in total citations were observed in 2009 and 2018. According to the above results, research interest in PCa immunotherapy has gradually increased over the years and has become a research hotspot.

### Analysis of the top 10 productive countries/regions, institutions, and authors showed that the USA is the leader in this field

**Figure 3:**
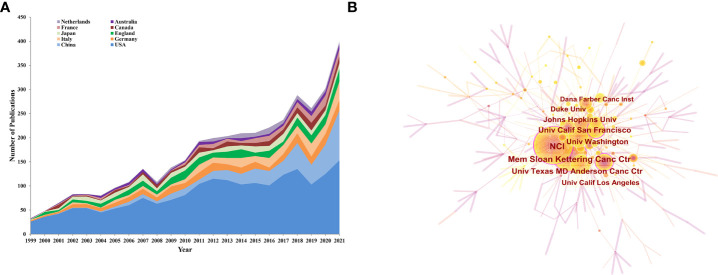
**(A)** The annual trend publications of the top 10 countries/regions related to PCa immunotherapy from 1999 to 2021. **(B)** The top 10 productive institutions on PCa immunotherapy-related literature.

For this part, we analyzed the top 10 productive countries/regions, institutions, and authors to identify leaders in this field. The trend for the number of annual publications related to PCa immunotherapy by the top 10 productive countries/regions from 1999 to 2021 is shown in **Figure 3A**, and the USA was the most productive country with 1,954 publications (54.54%), followed by China (12.50%, 448 publications), Germany (7.48%, 268 publications), Italy (7.26%, 260 publications), and England (6.89%, 247 publications) (**Table 1**). The cooperative relationship between different countries/regions was also analyzed. The country/region with the highest total link strength (TLS) was the USA, followed by England, which exhibited a close mutual cooperative relationship (**Figure S1A**).

**Table 1:** Top 10 productive countries/regions and organizations related to PCa immunotherapy research.

Rank	Country	Counts	Percentage	H-index	Total Citations	TLS	Institutions	Documents	Citations	TLS
1	USA	1,954	54.54%	145	107,849	817	NCI	185	10,978	318
2	China	448	12.50%	50	9,624	177	Memorial Sloan Kettering Cancer Center	137	18,581	424
3	Germany	268	7.48%	59	13,554	267	University of California San Francisco	102	10,955	343
4	Italy	260	7.26%	49	9,925	323	University of Texas MD Anderson Cancer Center	94	5,146	316
5	England	247	6.89%	56	10,485	335	Johns Hopkins University	93	16,166	298
6	Japan	196	5.47%	36	5,047	76	University of Washington	81	9,328	381
7	Canada	146	4.08%	42	6,299	172	University of California, Los Angeles	72	3,601	144
8	France	133	3.71%	48	7,395	262	Duke University	68	4,050	179
9	Australia	95	2.65%	33	5,110	116	Harvard University	65	8,705	230
10	Netherlands	94	2.62%	38	6,139	218	Dana-Farber Cancer Institute	62	12,064	306

TLS, total link strength.

The most active institutions are shown in **Figure 3B**, and more details are listed in **Table 1**. The National Cancer Institute (NCI) was the most productive institution with 185 publications and 10,978 citations, followed by the Memorial Sloan Kettering Cancer Center (137 publications with 18,581 citations), and the University of California San Francisco (102 publications with 10,955 citations). The top 10 productive institutions were all from the USA. Furthermore, the partnerships between institutions (**Figure S1B**) showed that the Memorial Sloan Kettering Cancer Center has close relationships with many institutions.

The top 10 prolific authors on PCa immunotherapy from 1999 to 2021 are listed in **Table 2**. Gulley, James L. (80 publications) has published the most papers, followed by Madan, Ravi A. (61 publications) and Drake, Charles G. (58 publications). Further analysis revealed that 8 of the top 10 authors were from the USA and the remaining 2 were from Japan. The top 10 co-cited authors with centrality are shown in **Table 2**, and Small, Eric J. was the most co-cited author, followed by Fong, Lawrence. **Figure S1C** is a visualization map of the top 100 co-cited authors with the highest TLS; Kantoff, P.W. was at the center of this field, followed by Small, Eric J.

**Table 2:** The 10 most productive authors and the top 10 co-cited authors with the highest centrality.

Rank	Author	Country	Counts	Total Citations	Co-Cited Author	Country	Total Citations	TLS	Centrality
1	Gulley, James L.	USA	80	3,732	Small, Eric J.	USA	1,176	85,963	0.24
2	Madan, Ravi A.	USA	61	1,687	Fong, Lawrence	USA	520	41,178	0.21
3	Drake, Charles G.	USA	58	5,377	Hodi, F. Stephen	USA	645	59,820	0.16	
4	Mcneel, Douglas G.	USA	50	1,629	Scher, Howard I.	USA	705	52,437	0.16
5	Fong, Lawrence	USA	45	2,892	Slovin, Susan F.	USA	495	42,379	0.16
6	Small, Eric J.	USA	44	4,160	Rosenberg, Steven A.	USA	738	78,555	0.15
7	Itoh, Kyogo	Japan	42	984	Tjoa, Benjiamin A.	USA	223	16,566	0.15
8	Schlom, Jeffrey	USA	40	2,573	Sanda, Martin G.	USA	227	16,230	0.12
9	Antonarakis, Emmanuel S.	USA	38	1,369	Nestle, Frank O.	USA	185	14,582	0.11
10	Noguchi, Masanori	Japan	34	838	Kwon, Eugene D.	USA	546	43,767	0.10

TLS, total link strength.

### Journals and co-cited journals analysis

**Figure 4:**
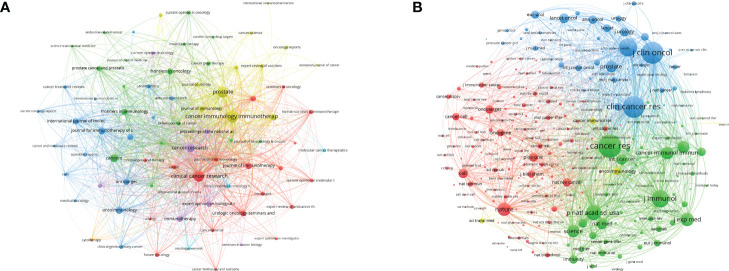
The visualization network of journals **(A)** and co-cited journals **(B)** related to PCa immunotherapy. The nodes with the same color represent the same cluster, implying a close partnership. The larger the node’s size or the width of the connecting line, the closer the relative degree of co-occurrence.

The identification of authoritative journals is convenient for researchers in related fields to understand current research trends and effectively track research hotspots. The network visualization of the most productive journals is shown in **Figure 4A**, and the top eight are listed in **Table S1** in detail. *Cancer Immunology Immunotherapy* was the most productive journal with 145 publications in the PCa immunotherapy field, followed by *Prostate* (*n* = 124 publications), *Clinical Cancer Research* (*n* = 122), *Cancer Research* (*n* = 86), and *Cancers* (*n* = 69). Of the top eight journals, the most cited was *Clinical Cancer Research* (*n* = 10,705), which was far more than other journals. All were classified as Q1/Q2 of Journal Citation Reports (JCR) in 2020, and the *Journal for Immunotherapy of Cancer* had the highest Impact Factor (IF) with 13.751. The network visualization of the most frequently co-cited journals is shown in **Figure 4B**. The top three with the largest nodes were *Cancer Research* (*n* = 11,945 citations), followed by *Journal of Clinical Oncology* (*n* = 11,090) and *Clinical Cancer Research* (*n* = 10,598). Detailed information on co-cited journals, such as country, IF, JCR, and total citations, is provided in **Table S1**. Of the top eight co-cited journals, the *New England Journal of Medicine* was the journal with the highest IF (91.245).

### Analysis of references and co-cited references revealed the fundamental studies and the most influential papers in this field

The most cited references are often considered the basis of research in a particular field. The top 10 original articles on PCa immunotherapy research are listed in **Table 3**. The article “Safety, activity, and immune correlates of anti-PD-1 antibody in cancer” from the *New England Journal of Medicine* by Topalian, Suzanne L., had the highest number of citations (*n* = 8,193), followed by “Sipuleucel-T immunotherapy for castration-resistant prostate cancer” (*n* = 3,629). The top three most cited references were all clinical trials. Almost all studies were published before 2010, except for the fifth and sixth studies.

**Table 3:** Top 10 original articles concerning the research of PCa immunotherapy.

Title	Journals	First author	Year	Citations
Safety, activity, and immune correlates of anti-PD-1 antibody in cancer	*New England Journal of Medicine*	Topalian, Suzanne L.	2012	8,193
Sipuleucel-T immunotherapy for castration-resistant prostate cancer	*New England Journal of Medicine*	Kantoff, Philip W.	2010	3,629
Phase I study of single-agent anti-programmed death-1 (MDX-1106) in refractory solid tumors: safety, clinical activity, pharmacodynamics, and immunologic correlates	*Journal of Clinical Oncology*	Brahmer, Julie R.	2010	2,033
Extrinsic versus intrinsic apoptosis pathways in anticancer chemotherapy	*Oncogene*	Fulda, S.	2006	1,556
Immune checkpoint targeting in cancer therapy: toward combination strategies with curative potential	*Cell*	Sharma, Padmanee	2015	1,305
Ipilimumab versus placebo after radiotherapy in patients with metastatic castration-resistant prostate cancer that had progressed after docetaxel chemotherapy (CA184-043): A multicentre, randomised, double-blind, phase 3 trial	*Lancet Oncology*	Kwon, Eugene D.	2014	941
Placebo-controlled phase III trial of immunologic therapy with sipuleucel-T (APC8015) in patients with metastatic, asymptomatic hormone refractory prostate cancer	*Journal of Clinical Oncology*	Small, Eric J.	2006	831
A phase I study on adoptive immunotherapy using gene-modified T cells for ovarian cancer	*Clinical Cancer Research*	Kershaw, Michael H.	2006	807
Biologic activity of cytotoxic T lymphocyte-associated antigen 4 antibody blockade in previously vaccinated metastatic melanoma and ovarian carcinoma patients	*Proceedings of the National Academy of Sciences of the United States of America*	Hodi, F.S.	2003	743
CTLA-4-mediated inhibition in regulation of T cell responses: Mechanisms and manipulation in tumor immunotherapy	*Annual Review of Immunology*	Chambers, C.A.	2001	724

**Figure 5:**
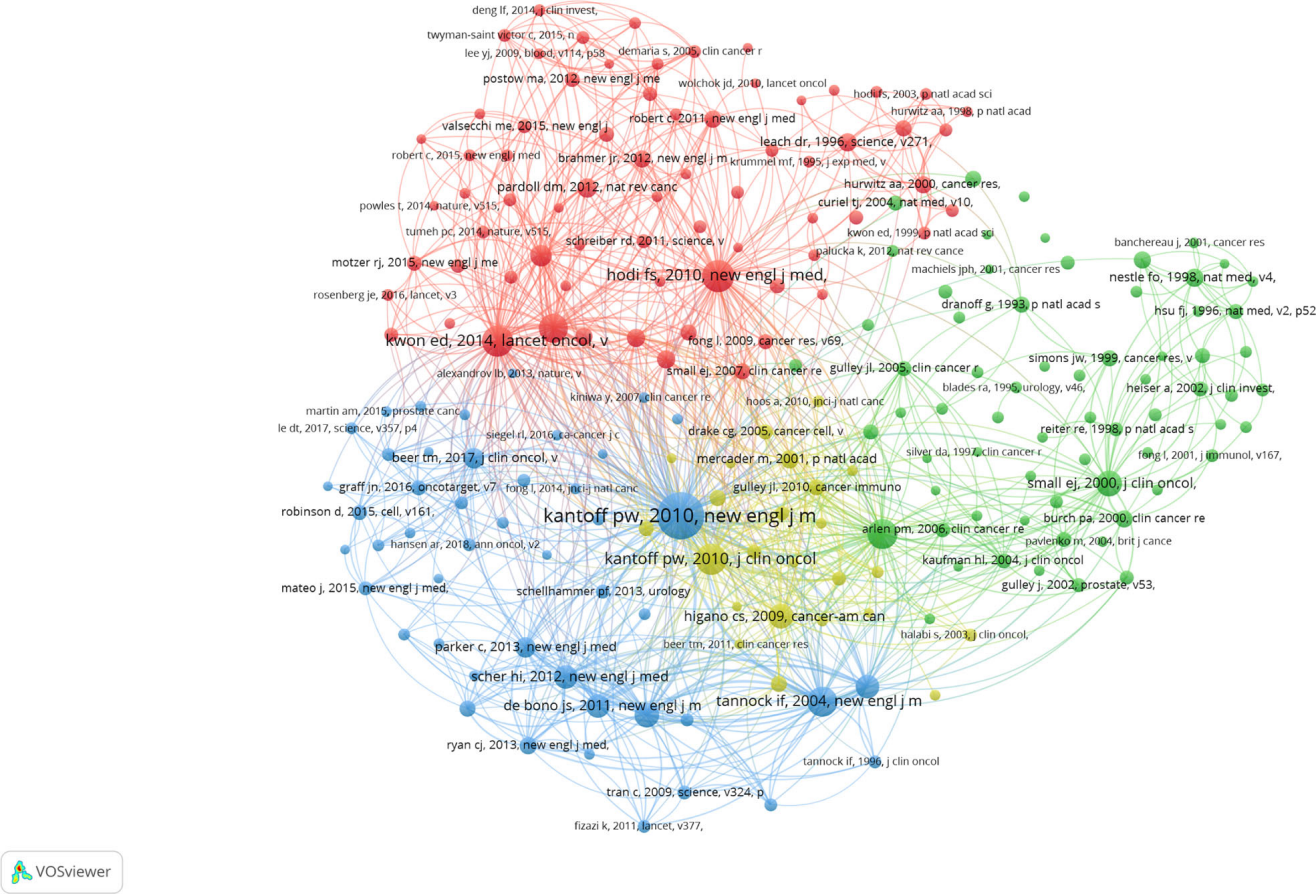
The visualization network of co-cited references in PCa immunotherapy.

**Figure 6:**
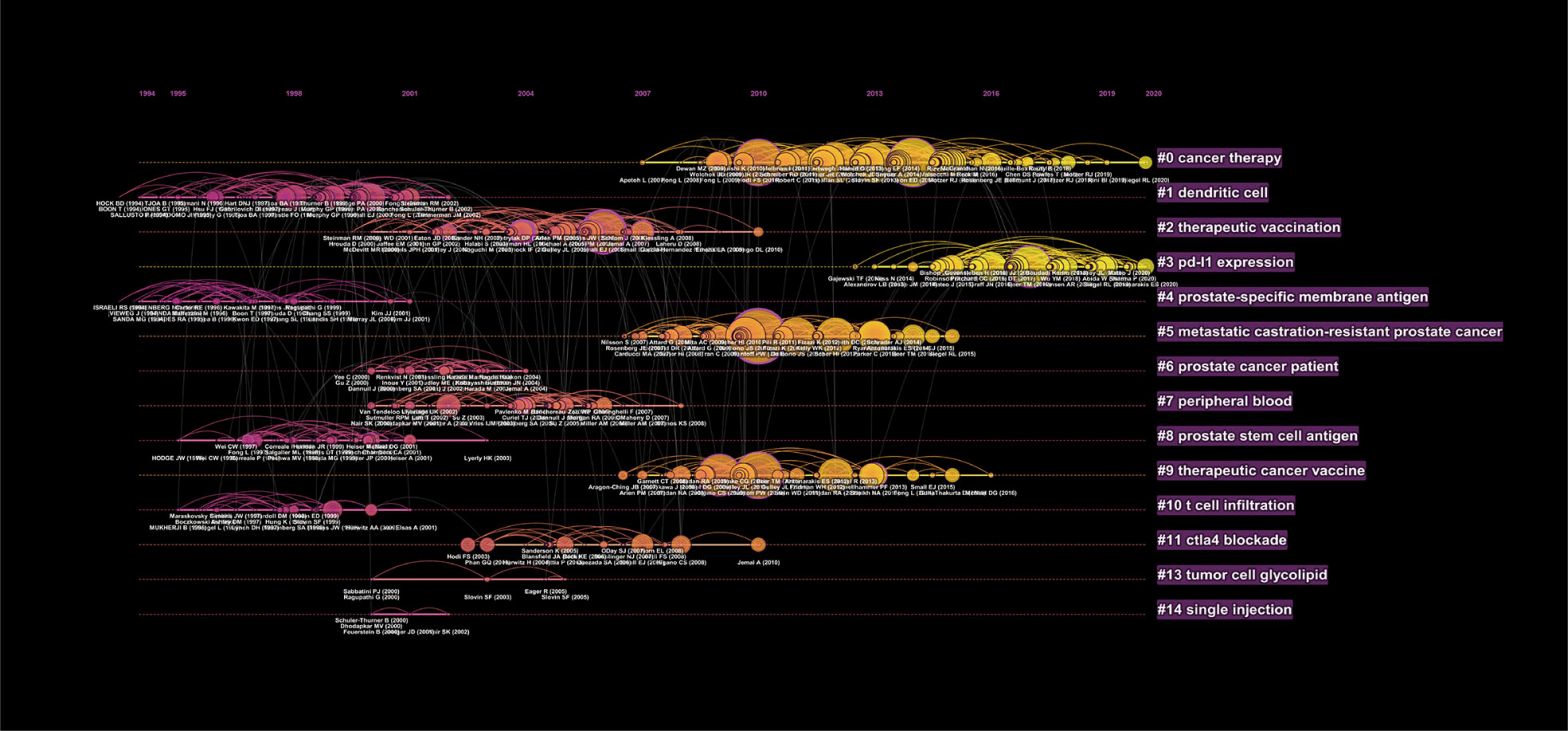
The timeline view of publications related to PCa immunotherapy with relevant clusters.

Meanwhile, a co-cited reference analysis was conducted in our study. The visualization network map is demonstrated in **Figure 5**, with more details in **Table 4**. As mentioned above, the article “Sipuleucel-T immunotherapy for castration-resistant PCa” by Kantoff, Philip. W. published in the *New England Journal of Medicine*, was the most cited in the top 10 co-cited references. We also analyzed the co-cited references through the timeline view (**Figure 6**). Indeed, we found that “PD-L1 expression” (Cluster 3) was a research hotspot in recent years. The clusters with larger nodes representing more citations indicated that “cancer therapy” (Cluster 0), “PD-L1 expression” (Cluster 3), “metastatic castration-resistant PCa” (Cluster 5), and “therapeutic cancer vaccine” (Cluster 9) were the hotspots in this field since 2007.

**Table 4:** Top 10 co-cited references involved in the research of PCa immunotherapy.

Title	First author	Year	Citations	Journals	IF (2020)
Sipuleucel-T immunotherapy for castration-resistant prostate cancer	Philip W Kantoff	2010	791	*New England Journal of Medicine*	91.245
Improved survival with ipilimumab in patients with metastatic melanoma.	F. Stephen Hodi	2010	408	*New England Journal of Medicine*	91.245
Ipilimumab versus placebo after radiotherapy in patients with metastatic castration-resistant prostate cancer that had progressed after docetaxel chemotherapy (CA184-043): A multicentre, randomised, double-blind, phase 3 trial	Eugene D. Kwon	2014	385	*Lancet Oncology*	41.32
Overall survival analysis of a phase II randomized controlled trial of a Poxviral-based PSA-targeted immunotherapy in metastatic castration-resistant prostate cancer	Philip W Kantoff	2010	367	*Journal of Clinical Oncology*	44.54
Docetaxel plus prednisone or mitoxantrone plus prednisone for advanced prostate cancer	Ian F Tannock	2004	347	*New England Journal of Medicine*	91.245
Placebo-controlled phase III trial of immunologic therapy with sipuleucel-T (APC8015) in patients with metastatic, asymptomatic hormone refractory prostate cancer	Eric J. Small	2006	344	*Journal of Clinical Oncology*	44.54
Safety, activity, and immune correlates of anti-PD-1 antibody in cancer	Suzanne L. Topalian	2012	335	*New England Journal of Medicine*	91.245
Immunotherapy of hormone-refractory prostate cancer with antigen-loaded dendritic cells	Eric J. Small	2000	274	*Journal of Clinical Oncology*	44.54
Integrated data from 2 randomized, double-blind, placebo-controlled, phase 3 trials of active cellular immunotherapy with sipuleucel-T in advanced prostate cancer	Celestia S Higano	2009	257	*Cancer*	6.860
Prednisone plus cabazitaxel or mitoxantrone for metastatic castration-resistant prostate cancer progressing after docetaxel treatment: A randomised open-label trial	Johann Sebastian de Bono	2010	251	*Lancet*	79.32

### Keyword analysis revealed the hot topics and research frontiers

**Figure 7:**
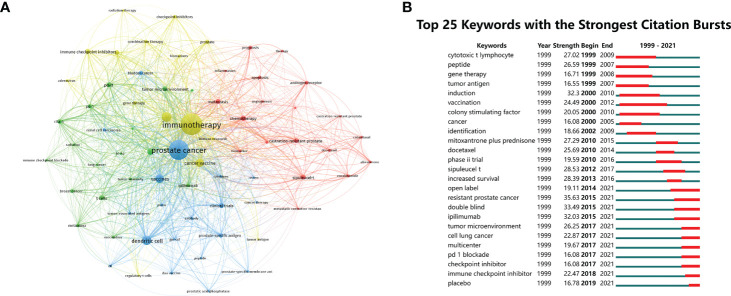
**(A)** The network visualization of author keywords. **(B)** Top 25 keywords with the strongest citation bursts in publications related to PCa immunotherapy.

To identify the hot topics in this field, keyword co-occurrence analysis, designed to clarify the co-occurrence relationship between keywords in publications, was performed. **Table 5** shows the top 20 co-occurrence keywords of PCa immunotherapy with the highest frequencies. The top 20 co-occurrence keywords were composed mainly of the terms “dendritic cell”, “vaccines”, and “cancer vaccine” related to tumor vaccine and the terms “PD-L1”, “immune checkpoint inhibitors”, and “CTLA-4” related to ICI. Meanwhile, author keywords network visualization is shown in **Figure 7A**, conducted by VOSviewer. All keywords were classified into four categories: cluster 1 (therapeutic approaches in PCa, red nodes), cluster 2 (mechanisms of tumor immunotherapy, green nodes), cluster 3 (tumor vaccines in PCa, blue nodes), and cluster 4 (ICI in PCa, yellow nodes).

**Table 5:** Top 20 co-occurrence keywords involved in the research of PCa immunotherapy.

Rank	Keywords	Frequency	TLS	Rank	Keywords	Frequency	TLS
1	immunotherapy	1,103	5,247	11	pd-l1	80	556
2	prostate cancer	954	4,297	12	tumor microenvironment	80	440
3	cancer	271	1,330	13	immune checkpoint inhibitors	78	414
4	dendritic cell	241	1,192	14	castration-resistant prostate cancer	76	351
5	vaccines	210	1,061	15	radiotherapy	75	403
6	cancer vaccine	191	1,016	16	sipuleucel-t	75	454
7	cancer immunotherapy	123	629	17	t cells	75	342
8	chemotherapy	91	534	18	prostate-specific antigen	73	524
9	clinical trials	85	515	19	ctla-4	66	403
10	metastasis	80	426	20	pd-1	62	530

TLS, total link strength.

The citation burst analysis of keywords was conducted by CiteSpace, involving burst strength and time of duration, which can reflect research hotspots in a certain period. The top 25 keywords with the strongest citation bursts were arranged by the beginning year of the burst from top to bottom, indicating the change in research trends for PCa immunotherapy from 1999 to 2021 (**Figure 7B**). The first keywords with a strong citation burst in this field began in 1999, including “cytotoxic t lymphocyte”, “peptide”, and “gene therapy”. The burst strength of “resistant PCa”, “double blind”, and “ipilimumab” was over 30 among these keywords. The keywords with the strongest citation burst in the past 5 years included “tumor microenvironment”, “multicenter”, “PD 1 blockade”, “checkpoint inhibitor”, “immune checkpoint inhibitor”, and “placebo”, suggesting that studies related to ICI are currently popular topics in PCa immunotherapy.

## Discussion

Immunotherapy, especially ICI, represents a new therapeutic approach for advanced PCa, which has demonstrated efficacy in delaying the malignancy progression in tumors with an active immune response (8–10). Therefore, the relationship between PCa and immunotherapy has attracted much interest in the past two decades. Regrettably, although significant progress has been achieved, ICI for PCa still has limitations when used for single-agent and combination therapy.

Bibliometric analysis has been widely used to clarify the status and follow the trends of research fields in recent years (31–33). To our knowledge, this is the first study to perform a systematic literature search for conducting the knowledge mapping and predicting the future research frontiers about PCa immunotherapy.

Based on the annual number of publications and citations shown in **Figure 2**, research interest in PCa immunotherapy has exhibited a steady upward trend. The three time points 2006–2007, 2010–2012, and 2017 were the moments of burst for publications and citations, which were accompanied by several major studies and events. In 2006–2007, the first phase III clinical trial about sipuleucel-T (APC8015) in PCa patients was published (34). After 3 years, another study indicated that sipuleucel-T prolonged OS among men with mCRPC (13); hence, sipuleucel-T was approved by the FDA for mCRPC patients in April 2010. In the same year, research about single-agent anti-PD-1 (MDX-1106) was also published, which involved various types of human tumors, including PCa (35). After 2017, an increased number of ICIs were approved by FDA for non-small cell lung cancer, melanoma, and urothelial carcinoma. Simultaneously, a series of studies were devoted to exploring the possibility of ICI *via* single-agent or combination therapy for PCa. For example, ICI significantly increased Th1 lineage and improved survival in the subcutaneous CRPC model but failed in the bone metastatic CRPC model since PCa promotes osteoclast-mediated bone resorption that releases TGF-β, which inhibits the development of Th1 lineage. Combined TGF-β neutralizing antibody and ICI could increase Th1 lineage, promote clonal expansion of CD8 T cells, and improve survival rate in the CRPC model of bone metastasis (36). Another study demonstrated the key role of CHD1 in MDSC recruitment and found that CHD1/IL6 is the main regulator of immunosuppressive TME in PTEN-deficient PCa. Interestingly, pharmacological inhibition of IL6 combined with ICI triggered a strong antitumor response in PCa (37).

In the present study, the USA is the most productive country in PCa immunotherapy. Cancer Statistics reported in 2021 by the American Cancer Society estimated that PCa ranked first and second in incidence and mortality for male malignant tumors in the USA (7). The incidence rate of PCa in the USA was higher than in other parts of the world, probably due to race, diet, lifestyle, and other factors, resulting in a heavy health burden (38). For this reason, many studies on PCa immunotherapy were conducted in the USA, accounting for 54.54% of the total publications. Meanwhile, the top 10 productive organizations related to this field were all from the USA, which corroborated its dominance in this field.

Next, we identified the influential experts in the field of PCa immunotherapy by the productive author list and the centrality of the co-cited author. Small, Eric J. from the USA had the largest centrality, with 44 publications and 1,176 citations. The clinical trial about sipuleucel-T (APC8015) (34) published in 2006 by him and his team was influential and attracted much attention. A comprehensive analysis of the most productive and co-cited journals in the field of PCa immunotherapy indicated that *Cancer Research* was the journal most co-cited by researchers in this field.

The analysis of the top 10 co-cited references, considered an indicator of significance reflected by the number of citations, is helpful for researchers to understand the important achievements in a particular field. The details of references are shown in **Table 4**, and most are clinical trials published in high-quality journals. We analyzed these documents and the significance behind them in the field of PCa immunotherapy according to the time axis.

In 2000, Small, Eric J. attempted to use antigen-loaded dendritic cells for hormone-refractory PCa immunotherapy. Provenge, the immunotherapy product consisting of autologous dendritic cells loaded *ex vivo* with a recombinant fusion protein, demonstrated good safety and effectiveness (39). Six years later, based on the encouraging results of phase I and phase II trials, a follow-up phase III trial (D9901) from the same expert was published in the *Journal of Clinical Oncology*, showing that sipuleucel-T may provide a survival advantage to asymptomatic metastatic hormone-refractory PCa patients (34). Subsequently, this research team conducted two phase III trials (D9901 and D9902A), demonstrating a survival benefit for PCa patients treated with sipuleucel-T compared to placebo patients (40). Finally, a double-blind, placebo-controlled, multicenter phase III trial of sipuleucel-T showed that it significantly prolonged OS among men with mCRPC (13). In April 2010, sipuleucel-T was approved by FDA for treating mCRPC patients. However, despite its good efficacy against PCa, the clinical usage of sipuleucel-T is limited due to lack of availability, the complexity of administration, and cost issues (41). Additionally, Philip W. Kantoff et al. also explored the efficacy of vaccine-related immunotherapy. PROSTVAC-VF immunotherapy, based on prostate-specific antigen (PSA)-targeted poxviral vaccines for PCa, was well tolerated and related to a 44% decrease in mortality and an 8.5-month improvement for median OS among men with mCRPC in a phase II trial (42). However, the phase III trial in 2019 indicated that PROSTVAC-VF did not affect the endpoints such as OS or alive without events (AWE) in mCRPC. Combination therapy is currently being explored in clinical trials (43).

ICI is another important approach for cancer immunotherapy. Immune checkpoints currently used for pharmaceutical development include CTLA-4 and PD-1/PD-L1. In 2010, immunotherapy based on immune checkpoints was first applied to melanoma. The two phase III trials by F. Stephen Hodi showed that it improved OS in patients with previously treated metastatic melanoma (44). For PCa, the effect of ipilimumab after radiotherapy on patients with mCRPC that progressed after docetaxel chemotherapy was assessed by Kwon, Eugene D., but there was no significant difference between the ipilimumab group and the placebo group in terms of OS during the primary analysis (14). Another ICI, nivolumab, was also applied for various cancer immunotherapies, including advanced melanoma, non-small cell lung cancer, castration-resistant PCa, renal cell cancer, or colorectal cancer. Regrettably, no objective responses were observed with PD-1 monotherapy in patients with colorectal cancer or PCa, considered as low immune response tumors (45). In addition, the combination of ipilimumab and nivolumab was used in a large trial for patients with mCRPC (published in *Cancer Cell*). The objective response rate was 25% in cohort 1 (pre-chemotherapy; *n* = 45) and 10% in cohort 2 (post-chemotherapy; *n* = 45) (16). However, the incidence of grade 3–4 adverse events was 42%–53% of patients, suggesting the necessity of optimizing the dose/schedule. Due to the limited clinical benefits of PD1/PD-L1 and CTLA-4, other immune checkpoints have been explored by researchers, such as the V-domain immunoglobulin suppressor of T-cell activation (VISTA), T-cell immunoglobulin domain and mucin domain 3 (TIM-3), and lymphocyte activation gene 3 (LAG-3) (46). For example, researchers found that ipilimumab therapy significantly increased VISTA expression on CD4 T cells, CD8 T cells, and CD68^+^ macrophages from matched pre- and post-treatment prostate tumors. VISTA is considered a compensatory inhibition pathway of ipilimumab in treating PCa, and combined treatment may bring meaningful clinical benefits (47).

Apart from understanding the research hotspots and changes in the development of PCa immunotherapy by co-cited references above, we can also objectively track the focus of publications and future development trends at different time points by analyzing the timeline view of references (**Figure 6**).

It has been reported that #1 dendritic cell, #2 therapeutic vaccination, #4 prostate-specific membrane antigen, and #9 therapeutic cancer vaccine could be clustered into cancer vaccines. Cancer vaccines are designed to activate the immune system to eliminate tumor cells. It has been established that mutations occur during tumor growth, accompanied by the production of new proteins (neoantigens), which could be recognized by immune cells. However, the intrinsic mechanism of tumor cells and the interaction with other cells effectively protect tumor from the immune system (48). Inspired by the effect of vaccines against pathogens, researchers manipulated tumor or immune cells (antigen-presenting cells or T cells) *in vitro* and then transfused them into the human body to activate the patient’s immune system. Except for sipuleucel-T (dendritic cell-based) and PROSTVAC-VF (PSA-based), GVAX is another vaccine indicated for PCa, consisting of inactivated PCa cell lines (PC-3, LNCaP) that could secrete GM-CSF and be effective in tumor antigen presentation. Two phase II trials of GVAX in asymptomatic metastatic CRPC showed effective antitumor activity, but the subsequent phase III trial was terminated at the interim analysis due to lack of efficacy. These tumor vaccines were well tolerated with infusion reactions or reversible influenza-like symptoms at the beginning of treatment (41, 49, 50).

PCa is an ideal candidate for cancer vaccine therapies, given its high targetable number of PSA and prostate-specific membrane antigen (PSMA) (51). Although PSMA has been studied for a long time (indicated by our timeline), cancer vaccine related to PCa based on PSMA has made encouraging progress in recent years (52). Lutetium-177 [^177^Lu]-PSMA-617, a radiolabeled small molecule, binds with high affinity to PSMA, enabling beta particle therapy targeting to mCRPC, showing high response rates, low toxic effects, and reduction of pain in patients with mCRPC who have progressed after conventional treatments (53).

Moreover, it has been reported that #0 Cancer therapy, #3 PD-L1 expression, and #11 CTLA-4 could be clustered into PCa immunotherapy by ICI. It is widely thought that mCRPC is an immunologically “cold” tumor due to a relatively low somatic mutation frequency and few tumor-infiltrating T cells, leading to resistance to immune checkpoint therapy (54). Higher tumor mutation burden (TMB) tumors, like melanoma and non-small lung cancer, with higher levels of neoantigens, are usually more responsive to immunotherapy. Accordingly, TMB can be applied as a biomarker to predict patient response to ICI. Although studies found that high expression of PD-L1, another biomarker of ICI response, was associated with a variety of clinical parameters, such as proliferation (Ki-67), Gleason Score, and androgen receptor expression (55), and was an independent biomarker in the prognosis of high-risk PCa patients who received adjuvant hormonal therapy after radical prostatectomy (56), the results of relevant clinical trials were unsatisfactory probably due to the tumor immunosuppressive microenvironment. It is widely acknowledged that the tumor immunosuppressive microenvironment is composed of multiple immunosuppressive cells [tumor-associated macrophages, myeloid-derived suppressor cells (MDSC), or regulatory T cells] and non-cellular components (chemokines, cytokines, or signaling molecules), resulting in less number or limited function of tumor-infiltrating T lymphocytes. Reversing the immunosuppressive microenvironment or increasing T lymphocyte infiltration is considered the main strategy to improve the effect of ICI on PCa. Meanwhile, emphasis has been placed on exploring other biomarkers that can predict the immunotherapy response for patients with PCa by whole-exome sequencing on tumor samples, which helps identify patients who can benefit from immunotherapy (57).

There were some limitations in this study. Indeed, it is well-established that English is the most global language, and most publications are in English. However, it should be borne in mind that there are still some important studies published in other languages that were not included in this study. Moreover, only publications from WoSCC were included in this study, which may cause selection bias. Next, the bibliometric analysis relies heavily on the number of publications and total citations, but it takes time to accumulate citations. Our results showed that most of the highly cited articles were published 5 years before, and many publications that did not appear in our analysis may also be highly influential. Therefore, it is necessary to update and track in a timely manner.

## Conclusion

In this study, we obtained a comprehensive overview and potential directions of PCa immunotherapy by bibliometric analysis. Exploring complex immunogenicity and TME for PCa helps provide novel insights for reversing the immunosuppressive microenvironment and identifying the patient populations who can benefit from immunotherapy. Combining ICI with other therapeutic options and cancer vaccines represents the future development trends in PCa immunotherapy.

## Data availability statement

Publicly available datasets were analyzed in this study. This data can be found here: https://www.webofscience.com/wos/alldb/basic-search.

## Author contributions

WW, JK, and XM conceived and designed the study. WZ and ZS collected the data and made data visualization. WZ and YW drafted and revised the article. All authors contributed to the article and approved the submitted version.

## Funding

This research was supported by grants from the National Natural Science Foundation of China (Grant No. 82173039).

## Acknowledgments

The authors thank Dr. Yukun Wu for his effort in polishing the English content of this manuscript.

## Conflict of interest

The authors declare that there were no any commercial or financial relationships that could be construed as a potential conflict of interest.

## Publisher’s note

All claims expressed in this article are solely those of the authors and do not necessarily represent those of their affiliated organizations, or those of the publisher, the editors and the reviewers. Any product that may be evaluated in this article, or claim that may be made by its manufacturer, is not guaranteed or endorsed by the publisher.

## Abbreviations

ADT, androgen deprivation therapy; AWE, alive without events; CAR, chimeric antigen receptor; CTLA-4, cytotoxic T lymphocyte antigen 4; FDA, Food and Drug Administration; ICI, immune checkpoint inhibitor; IF, Impact Factor; JCR, Journal Citation Reports; mCRPC, metastatic castration-resistant prostate cancer; MDSC, myeloid-derived suppressor cells; NCI, National Cancer Institute; OS, overall survival; PARP, poly ADP-ribose polymerase; PCa, prostate cancer; PD-1, programmed cell death protein 1; PD-L1, programmed cell death ligand 1; PSA, prostate-specific antigen; PSMA, prostate-specific membrane antigen; TGF, transforming growth factor; TLS, total link strength; TME, tumor microenvironment; TMB, tumor mutation burden; USA, United States of America; WoSCC, Web of Science Core Collection.
