# Supplementary material for: Knowledge mapping and current trends of immunotherapy for prostate cancer: A bibliometric study
Source: Front Immunol. 2022 Oct 27;13:1014981. doi: 10.3389/fimmu.2022.1014981 (PMC9647028; doi:10.3389/fimmu.2022.1014981)
Supplement: Supplementary file 1 [file DataSheet_1.docx]

Supplementary Material

1. **Supplementary Figures and Tables**

**1.1 Supplementary Figures**

**
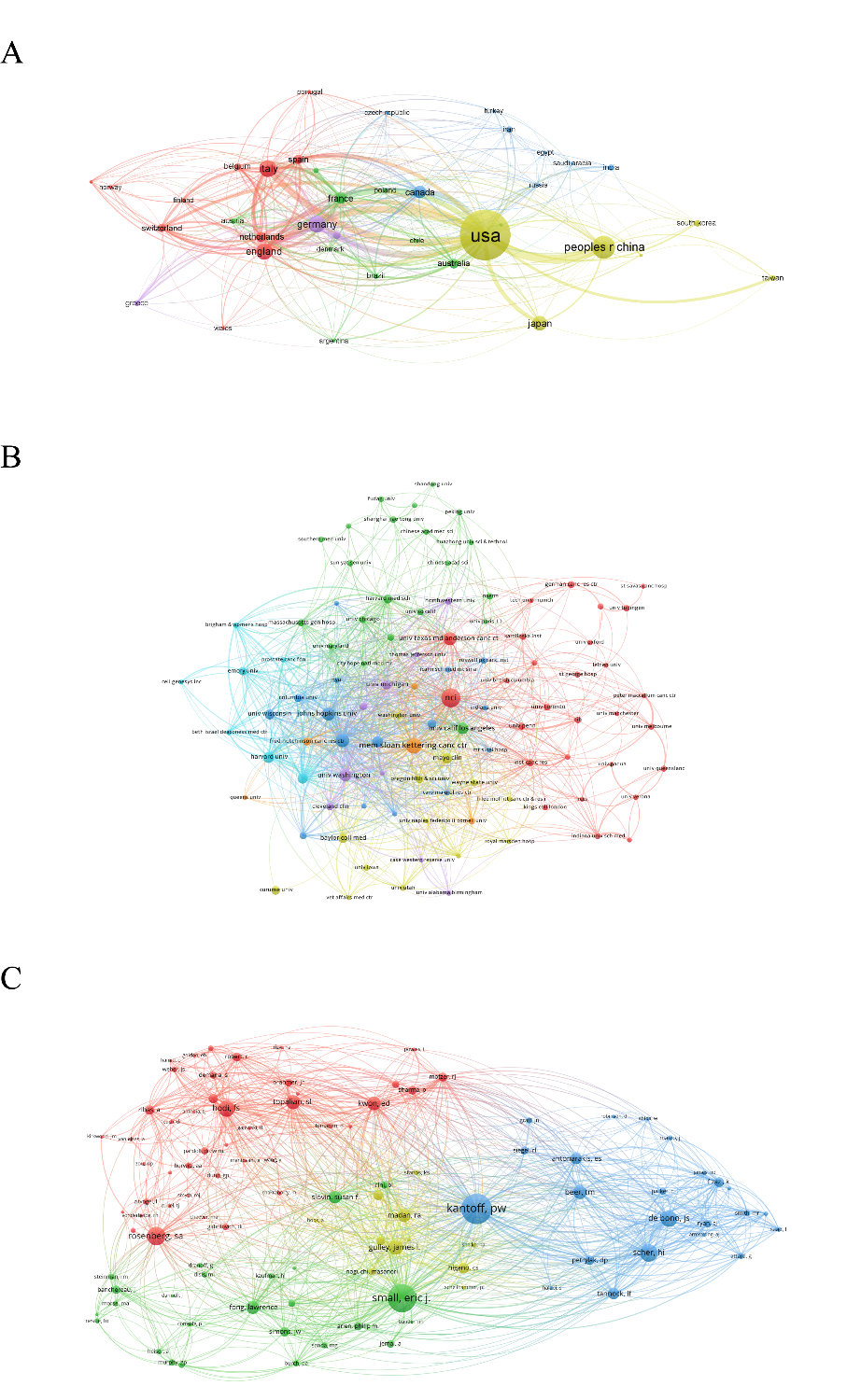
**

**Supplementary Figure S1** The visualization network of the cooperative partnerships between countries/regions (A), institutions (B), and authors (C) related to PCa immunotherapy conducted by VOSviewer.

## 1.2 Supplementary Tables

**Table S1. Top 8 productive journals and co-cited journals in the field of PCa immunotherapy.**

| Rank | Journals | Country | Count | IF  (2020) | JCR  (2020) | H-index | Total citations | Co-cited journals | Country | IF  (2020) | JCR  (2020) | Total citations |
| --- | --- | --- | --- | --- | --- | --- | --- | --- | --- | --- | --- | --- |
| 1 | Cancer Immunology Immunotherapy | Germany | 145 | 6.968 | Q1 | 41 | 4986 | Cancer Research | USA | 12.701 | Q1 | 11945 |
| 2 | Prostate | USA | 124 | 4.104 | Q2 | 33 | 3777 | Journal of Clinical Oncology | USA | 44.544 | Q1 | 11090 |
| 3 | Clinical Cancer Research | USA | 122 | 12.531 | Q1 | 58 | 10705 | Clinical Cancer Research | USA | 12.531 | Q1 | 10598 |
| 4 | Cancer Research | USA | 86 | 12.701 | Q1 | 48 | 6713 | New England Journal of Medicine | USA | 91.245 | Q1 | 7923 |
| 5 | Cancers | Switzerland | 69 | 6.639 | Q1 | 17 | 753 | Journal of Immunology | USA | 5.422 | Q2 | 7332 |
| 6 | Journal for Immunotherapy of Cancer | UK | 57 | 13.751 | Q1 | 21 | 1474 | Proceedings of The National Academy of Sciences of The United States of America | USA | 11.205 | Q1 | 5669 |
| 7 | Oncoimmunology | USA | 55 | 8.11 | Q1 | 22 | 1836 | Journal of Experimental Medicine | USA | 14.307 | Q1 | 4523 |
| 8 | Journal of Immunotherapy | USA | 48 | 4.456 | Q2 | 25 | 2382 | Nature | UK | 49.962 | Q1 | 4440 |
